# P04-07 Factors associated with maintenance of physical activity in older adults undertaking a strength and balance programme for falls prevention

**DOI:** 10.1093/eurpub/ckac095.061

**Published:** 2022-08-29

**Authors:** Claire Blackmore, Denise Kendrick, Elizabeth Orton

**Affiliations:** 1 University of Leicester, Leicester, United Kingdom; 2 University of Nottingham, Nottingham, United Kingdom

**Keywords:** strength, balance, falls, older adults

## Abstract

**Background:**

Falls are a major cause of mortality and morbidity in older adults worldwide, yet those who are more physically active have a lower risk of falling. There is little information on which participants are most likely to complete falls prevention exercise programmes and increase their levels of physical activity (PA). This study aims to identify factors associated with completion of, and PA levels, at the end of the Falls Management Exercise (FaME) falls prevention exercise programme, a programme designed to increase balance and functional capacity, increase bone and muscle mass and reduce fear of falling.

**Methods:**

356 community-dwelling adults provided routine data. Characteristics of participants were compared at baseline. Comparison of activity levels between completers and non-completers were carried out, and a regression analysis performed to identify factors associated with programme completion and achievement of 150 minutes of moderate to vigorous physical activity (MVPA) per week.

**Results:**

143 participants completed the FaME programme. This group was significantly younger (range 50-96; mean=75.3, *SD*=8.1 in completers *vs.* mean=77.8, *SD*=8.3 in non-completers) and had significantly lower scores on the FRAT (median=1, IQR=0-2 in completers *vs.* median=2, IQR=1-3 in non-completers) and FES-I risk assessments (median=10, IQR=7-13 in completers *vs.* median=11, IQR=8-16 in non-completers) at baseline, and a significantly higher level of physical activity (PA) per week (median=673 minutes, IQR=252-1252 in completers *vs.* median=558, IQR=120-1127 in non-completers). Completers significantly increased their total minutes of PA per week, and the converse was true for non-completers. Multivariate regression analyses showed that FRAT score was significantly associated with completion of FaME, and 180 degree turn and FES-I score were significantly associated with achieving the recommended 150 minutes of MVPA per week.

**Conclusions:**

This study has shown that a significant increase in PA levels is only demonstrated in those who complete the FaME programme. Scores from selected functional measures and risk assessments are associated with FaME completion and achievement of 150 minutes of MVPA. This information can be used to provide targeted support to improve completion rates and physical activity levels of participants.

